# A Thermodynamic Framework for Predicting Oxygen Vacancy Formation Energies on Electrocatalyst Surfaces

**DOI:** 10.1021/acsomega.6c03286

**Published:** 2026-06-10

**Authors:** Yuefeng Zhang, Zhenbin Wang

**Affiliations:** 1 Department of Materials Science and Engineering, 53025 City University of Hong Kong, Hong Kong SAR 999077, China; 2 School of Energy and Environment, 53025 City University of Hong Kong, Hong Kong SAR 999077, China

## Abstract

Oxygen vacancies (V_O_) are widely invoked as key descriptors of the activity of transition metal oxide electrocatalysts. However, their thermodynamic stability under realistic electrochemical operating conditions remains poorly understood. In this work, we establish a thermodynamic framework for calculating V_O_ formation energies as a function of pH and applied potential. Through a systematic evaluation of 19 representative oxygen reduction reaction (ORR) and evolution reaction (OER) catalysts, we demonstrate that while V_O_ formation can be energetically favorable at standard states (0 V vs RHE), the formation energies increase significantly under operando conditions, exceeding 0.50 eV for ORR and 2.00 eV for OER. These large thermodynamic barriers suggest that surface oxygen vacancies are unlikely to persist as stable, long-lived surface defects during electrocatalysis, particularly under the oxygen-rich OER conditions. Our findings challenge the prevailing assumption of vacancy-mediated reaction mechanisms and highlight the necessity of accounting for potential-dependent surface stoichiometry when modeling the catalyst–electrolyte interface.

## Graphical abstract

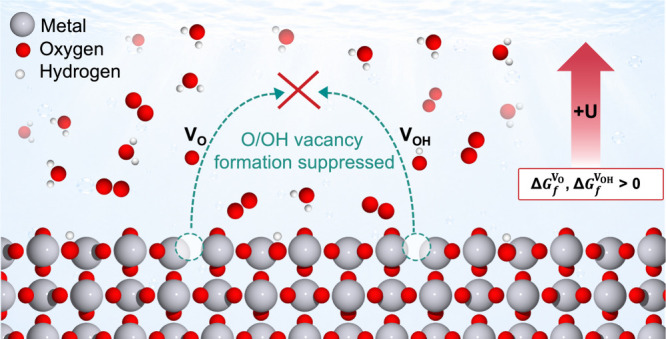

## Introduction

Oxygen electrocatalysis, specifically the oxygen reduction reaction (ORR) and oxygen evolution reaction (OER), is fundamental to sustainable energy conversion and storage technologies such as fuel cells, electrolyzers, and metal-air batteries. Within these processes, oxygen vacancies (V_O_) are recognized as critical factors that govern ionic conductivity, electronic structure, and surface reactivity.^1,2^ Consequently, quantifying the vacancy formation energy is essential for accurately modeling reaction mechanisms and understanding catalyst performance. However, experimental determination of these energies remains challenging due to the indirect nature of measurements, sensitivity to working environments, and the difficulty of distinguishing surface defects from bulk vacancies.^3^ While density functional theory (DFT) calculations have become an invaluable tool for evaluating these properties across charge states and pressures,^4–6^ a well-established framework for assessing vacancy formation specifically under electrocatalytic conditions, where pH and applied potential dictate stability, is still lacking.

Many existing studies^7–11^ interpret electrochemical behavior by calculating the catalytic activity of surfaces presumed to contain oxygen vacancies, thereby implicitly assuming their presence under operating conditions. Although this approach has achieved some agreement between experimental observations and computational predictions, neglecting the thermodynamic feasibility of vacancy formation under bias may undermine the validity of proposed mechanisms. Without accounting for the stability of these defects under operando conditions, interpretations of catalytic properties risk being based on surface structures that are fundamentally unstable in oxygen-rich or high-potential environments.

In this work, we establish a general thermodynamic framework for calculating vacancy formation energies under electrochemical operando conditions by incorporating pH and applied potential dependencies. Using this methodology, we systematically investigated the stability of oxygen vacancy (V_O_) in 19 representative oxygen electrocatalysts under relevant operating conditions. Our findings reveal that while V_O_ formation can be energetically favorable at standard state (0 V vs RHE), the formation energies increase significantly under realistic operating environments, exceeding 0.50 eV for ORR and 2.00 eV for OER. These large thermodynamic barriers indicate that surface oxygen vacancies are unlikely to persist as stable, long-lived surface defects during electrocatalysis, particularly under the oxygen-rich OER conditions. Ultimately, this operando framework challenges the prevailing assumption of surface vacancy stability, providing a more rigorous foundation for evaluating defect-mediated reaction mechanisms.

## Results

Figure 1a shows the calculated Δ*G*_f_^V_O_^ values for 18 representative oxygen electrocatalysts under standard state conditions (*U*_RHE_ = 0). These standard-state values are frequently cited in the literature as theoretical indicators of vacancy formability. A positive Δ*G*_f_^V_O_^ indicates that V_O_ formation is endothermic and thermodynamically unfavorable, whereas a negative value signifies spontaneous formation. Among the materials studied, NiFe_2_O_4_ (−1.02 eV), CoFe_2_O_4_ (−0.84 eV), SrCoO_3_ (−0.62 eV), LaNiO_3_ (*Pm* 3̅ m) (−0.56 eV), LaNiO_3_ (R 3̅ c) (−0.25 eV), LaCuO_3_ (R 3̅ c) (−0.19 eV) and β-MnO_2_ (0.10 eV) exhibit either negative or minimal positive formation free energies. This suggests that V_O_ formation in these materials is energetically facile under standard conditions and that they likely possess intrinsic vacancy concentrations. Conversely, LaCuO_3_ (*Pm* 3̅ m) (0.38 eV), γ-MnO_2_ (0.54 eV), β-NiOOH (0.59 eV), β-NiOOH:Fe (0.72 eV), Co_3_O_4_ (1.05 eV), PrBaCo_2_O_6_ (1.40 eV), CeO_2_ (1.87 eV), β-CoOOH (2.34 eV), IrO_2_ (3.25 eV), RuO_2_ (3.45 eV) and Fe_3_O_4_ (3.46 eV) show high positive values, indicating that vacancy formation in these materials is thermodynamically unfavorable under standard conditions.

**Figure 1:**
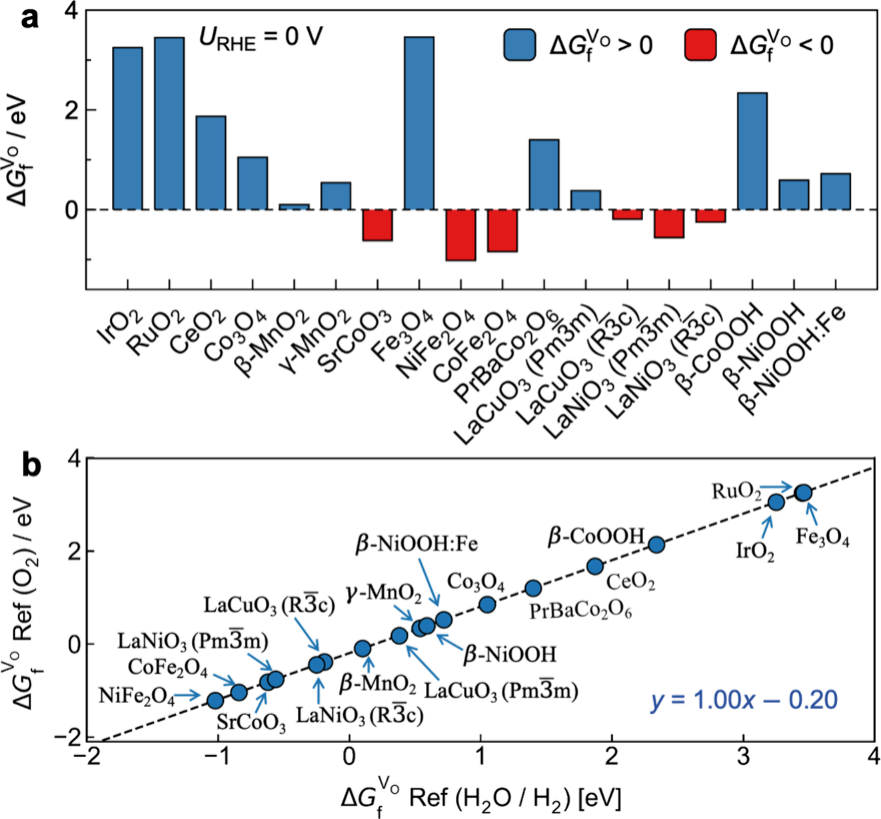
(a) Calculated oxygen vacancy (V_O_) formation free energies (Δ*G*_f_^V_O_^) for 18 metal oxides and 3*d* transition metal hydroxides under standard state conditions, where *U*_RHE_ = 0. γ-FeOOH was excluded because its surface is fully covered by OH species. (b) Linear scaling relationship between Δ*G*_f_^V_O_^ ref (O_2_) and Δ*G*_f_^V_O_^ ref (H_2_O/H_2_).

Given that the catalyst surface may deviate from its pristine state under working conditions, we further evaluated the influence of surface coverage on V_O_ formation. O-covered IrO_2_ and OH-covered LaCuO_3_ (R 3̅ c) were selected as representative covered surfaces. The calculated V_O_ formation energies are 3.01 eV and −0.19 eV, respectively, which are only slightly lower than or identical to those on the corresponding bare surfaces (3.25 eV and −0.19 eV). These results indicate that surface coverage has at most, a minor effect on V_O_ formation. In addition, we examined the effect of reaction intermediates on V_O_ formation using IrO_2_ (Figure S1). The calculated V_O_ formation energies are 3.05, 3.20, and 3.22 eV for the O*-, OH*-, and OOH*-covered surfaces, respectively, which are only 0.20, 0.05, and 0.03 eV lower than that of the bare surface (3.25 eV). These small decreases indicate that reaction intermediates have only a minor effect on V_O_ formation.

A notable distinction exists between formation energies calculated using an electrochemical reference versus the gas-phase O_2_ reference often found in thermal catalysis literature. Our evaluation shows that referencing gas-phase O_2_ results in a constant downward shift of 0.20 eV relative to electrochemical referencing, as shown in Figure 1b and Figure S2. This shift reflects a systematic offset associated with the choice of oxygen reference state, most likely arising from the well-known DFT-PBE error in describing the O_2_ binding energy.^12^ Additionally, using the corrected O_2_ reference^13^ increases the calculated V_O_ formation energies by ∼ 0.4 eV relative to electrochemical referencing.

To assess vacancy stability under realistic operating environments, we calculated Δ*G*_f_^V_O_^ for 15 oxides at typical ORR (*U*_RHE_ = 0.80 V) and OER (*U*_RHE_ = 1.60 V) potentials. As shown in Figure 2a, at *U*_RHE_ = 0.80 V, Δ*G*_f_^V_O_^ values for all 15 metal oxides are positive, with the lowest values recorded for NiFe_2_O_4_ (0.58 eV) and CoFe_2_O_4_ (0.76 eV). At *U*_RHE_ = 1.60 V (Figure 2b), these values become significantly more positive and are uniformly shifted upward by 1.6 eV relative to those at *U*_RHE_ = 0.80 V, with NiFe_2_O_4_ and CoFe_2_O_4_ increasing to 2.18 and 2.36 eV, respectively. This uniform potential-induced increase preserves the relative trend among different materials. These results indicate that V_O_ formation is increasingly difficult under ORR and OER conditions compared to the standard state. In particular, it is thermodynamically unlikely for V_O_ to form on electrocatalyst surfaces under OER working conditions. This is consistent with the physical expectation that V_O_ formation should be suppressed in oxygen-rich environment, where the oxygen chemical potential is high. The detailed Δ*G*_f_^V_O_^ as a function of applied potential (*U*_RHE_) for all above oxides are provided in Figure S3.

**Figure 2:**
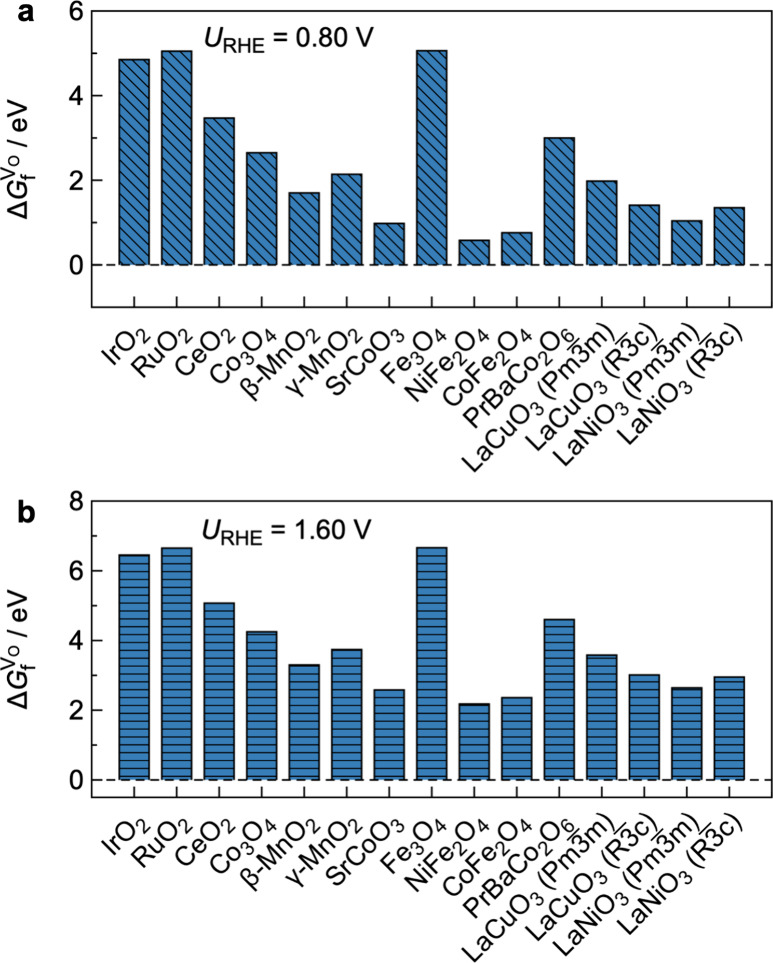
Calculated Δ*G*_f_^V_O_^ values for 15 metal oxides at applied potentials of (a) *U*_RHE_ = 0.80 V, and (b) *U*_RHE_ = 1.60 V.

The formation energetics for oxygen (V_O_) and hydroxyl (V_OH_) vacancies in 3d transition metal hydroxides were also evaluated (Figure 3). As illustrated in Figure 3a, the metal hydroxides possess a layered structure with van der Waals and hydrogen bonding interactions. β-CoOOH surface was asymmetric termination with OH groups on top surface and O groups on the bottom surface. β-NiOOH surface exhibited mixed termination with OH and O groups coexisting on top and bottom surfaces. γ-FeOOH surface exhibited symmetric surface termination with OH groups uniformly covering both top and bottom surfaces. At *U*_RHE_ = 0.80 V (Figure 3b), the calculated Δ*G*_f_^V_OH_^(Δ*G*_f_^V_O_^ in parentheses) for γ-FeOOH, β-NiOOH, β-NiOOH: Fe, and β-CoOOH are 1.75 eV, 1.91 eV (2.19 eV), 2.11 eV (2.32 eV), and 3.01 eV (3.94 eV), respectively. These large positive values suggest that neither vacancy type is likely to form under ORR condition. At *U*_RHE_ = 1.60 V (Figure 3c), these energies rise further, reaching 2.55 eV, 2.71 eV (3.79 eV), 2.91 eV (3.92 eV), and 3.81 eV (5.54 eV), respectively. The data consistently show that V_O_ formation is thermodynamically more challenging than V_OH_ formation in these hydroxide systems, and both are highly unfavorable under operando electrochemical conditions. The detailed Δ*G*_f_^V_OH_^(Δ*G*_f_^V_O_^) values as a function of applied potential (*U*_RHE_) for all these hydroxides are provided in Figure S4.

**Figure 3:**
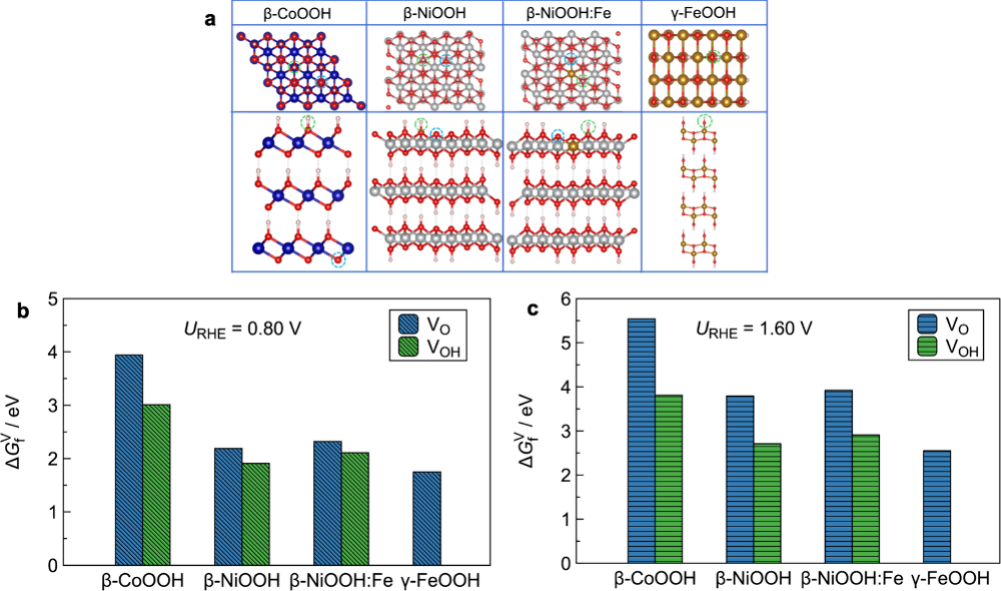
(a) Surface structural models of 3d transition metal hydroxides. The blue and green dashed circles indicate the locations of oxygen (O) and hydroxide (OH) vacancies, respectively. Calculated oxygen and hydroxyl vacancy formation free energies ( $\Delta {G_{f}}^{V}$ ) for 3d transition metal hydroxides at (b) *U*_RHE_ = 0.80 V and (c) *U*_RHE_ = 1.60 V. For γ-FeOOH, only Δ*G*_f_^V_OH_^ was calculated due to the absence of oxygen sites.

## Discussion

Numerous studies attribute enhanced ORR and OER activity to the presence of oxygen vacancies.^14–20^ Prominent examples include spinel-structured catalysts, such as Co_3_O_4_, CoFe_2_O_4_, and NiFe_2_O_4_. For instance, Rong et al.^21^ attributed the exceptional OER activity of vacancy-rich Co_3_O_4_ hollow nanotubes (265 mV at 10 mA cm^–2^) to strategically introduced oxygen vacancies, which were proposed to optimize the adsorption and desorption energy barriers of reaction intermediates. However, our results indicate that the oxygen vacancy formation energies in Co_3_O_4_ are as high as 4.25 eV under OER conditions, making it thermodynamically unlikely for such vacancies to form on the catalyst surface during operation. *This discrepancy raises a critical question: why are oxygen vacancies so frequently invoked to explain improved catalytic activity?*

The prevalence of this explanation likely stems from several factors. First, oxygen vacancies are often intentionally introduced during catalyst synthesis. Second, experimental characterization of these vacancies is typically conducted *ex situ* under ambient conditions rather than *in situ* under operating conditions. Similarly, computational studies often rely on DFT-calculated vacancy formation energies evaluated at standard states (see Figure 1), which do not accurately reflect electrocatalytic environments. While one might argue that vacancies introduced during synthesis could be kinetically stabilized, growing evidence^22^ from *in situ* experiments suggests that oxygen electrocatalysts, particularly those used for the OER, undergo significant dynamic surface reconstruction. These reconstruction processes facilitate the equilibration of kinetically trapped vacancies with the surrounding environment, a process ultimately governed by thermodynamics. Consequently, our results suggest that any claims linking enhanced ORR or OER activity to oxygen vacancies must, at the very least, be rigorously evaluated against formation energies calculated under realistic operating conditions, as presented in this work, or verified through comprehensive *in situ* surface characterization.

We note that this work is rooted in thermodynamic analysis and does not explicitly account for full reaction kinetic effects or dynamic surface reconstruction. Consequently, our results do not preclude the transient existence of surface oxygen vacancies under nonequilibrium operation. Nevertheless, the large thermodynamic driving force against V_O_ formation, together with *ab initial* molecular dynamics simulations (Figure S5), indicates that such vacancies are unlikely to accumulate as long-lived surface species, particularly under the oxygen-rich, high-potential conditions relevant to the OER. A more complete description of these nonequilibrium processes would require explicit treatment of surface restructuring, which represents an important direction for future work.

## Conclusion

In summary, we have established a general thermodynamic framework that accounts for pH and applied potential to evaluate the stability of oxygen vacancies under electrochemical conditions. Our systematic assessment of 19 representative oxygen electrocatalysts demonstrates that surface oxygen vacancies and hydroxyl vacancies are thermodynamically prohibitive under practical ORR and OER operating conditions. Specifically, the formation free energies exceed 0.50 and 2.00 eV for ORR and OER, respectively, indicating that surface stoichiometric recovery is strongly favored over defect persistence in oxygen-rich or high-potential environments.

These findings suggest that the enhanced catalytic activities often attributed to oxygen vacancies in the literature likely arise from other factors, such as synthesis-induced changes in bulk conductivity or surface reconstruction. Alternatively, they may reflect kinetically trapped vacancy states that are unlikely to persist under long-term operando conditions. Our results underscore that current vacancy-mediated reaction mechanisms must be re-evaluated using potential-dependent surface models to ensure thermodynamic consistency. This work provides a rigorous foundation for future computational and experimental studies, shifting the focus toward a more accurate, operando-based understanding of the catalyst–electrolyte interface to accelerate the rational design of high-performance electrocatalysts.

## Computational Methods

### Density Functional Theory (DFT) Calculations

Spin-polarized DFT calculations were performed using the Vienna *Ab initio* Simulation Package (VASP).^23^ The Perdew–Burke–Ernzerhof (PBE) functional^24^ was employed to describe exchange-correlation interactions. To mitigate self-interaction errors, the DFT+U method was applied with Hubbard U parameters of 3.90, 3.32, 5.30, 6.20, and 5.00 eV for Mn, Co, Fe, Ni, and Ce, respectively. These values were chosen to ensure an accurate description of redox reactions and are consistent with the Materials Project^25^ settings. Long-range van der Waals interactions in 3d transition metal hydroxides were accounted for using Grimme’s DFT-D3 empirical correction scheme.^26^ The electronic total energy convergence criterion was set to 10^–5^ eV. For bulk material calculations, an energy cutoff of 520 eV and an atomic force convergence threshold of 0.02 eV/Å were used. For surface calculations, the energy cutoff and atomic force convergence criteria were set to 450 and 0.05 eV/Å, respectively. The Brillouin zone was sampled using the Gamma scheme with a reciprocal *k*-point density of 100 Å^–3^. A vacuum spacing of at least 15 Å was introduced along the *z*-direction to prevent periodic interactions. The surface model for each material was constructed with a slab thickness of at least 10 Å, with the bottom half of the slab fixed at their bulk positions. A ferromagnetic configuration was adopted for all calculations, except for CoFe_2_O_4_ and NiFe_2_O_4_, where the ferrimagnetic configuration was employed, which was reported to be the most stable magnetic configurations in previous studies.^27^ *Ab initio* molecular dynamics simulations were carried out at the Γ point within the framework of Born–Oppenheimer dynamics as implemented in the VASP code.^28^ The slow-growth approach was applied to an explicitly solvated RuO_2_ model containing 27 H_2_O molecules to monitor the lattice-oxygen spillover process, and the corresponding free-energy barrier was evaluated using the Blue Moon ensemble method.^29^

### Materials and Model Selection

In this work, we selected 19 materials, including 15 oxides (RuO_2_, IrO_2_, β-MnO_2_, γ-MnO_2_, Fe_3_O_4_, Co_3_O_4_, CeO_2_, LaCuO_3_ (*Pm* 3̅ m), LaCuO_3_ (R 3̅ c), LaNiO_3_ (*Pm* 3̅ m), LaNiO_3_ (R 3̅ c), NiFe_2_O_4_, CoFe_2_O_4_, SrCoO_3_, PrBaCo_2_O_6_) and four oxyhydroxides (β-CoOOH, β-NiOOH, β-NiOOH:Fe, γ-FeOOH), to calculate the oxygen vacancy (V_O_) formation energy under relevant electrochemical operating conditions. The most stable surface of each material was used, and a large supercell slab model was constructed for each surface to simulate a dilute V_O_ concentration. Detailed model parameters are provided in Table 1 and all structure files are shared on Figshare. Notably, the crystal structure of β-NiOOH was obtained from ref 30, where it was generated by adding H to an orthorhombic NiOO*H*_1/2_ structure. We find that r2SCAN calculations, whether including the Hubbard U correction and/or van der Waals interactions (rVV10), predict this structure to be more stable than the two-formula-unit triclinic crystal structure for which PBE+U predicts ground state.^30^ All other crystal structures were obtained from the inorganic crystal structure database.^31^ The cubic LaCuO_3_ structure was generated by substituting the corresponding elements into a cubic perovskite structure. LaCuO_3_ (*Pm* 3̅ m) and LaNiO_3_ (*Pm* 3̅ m) are hypothetical phases frequently employed in reaction mechanism studies in the literature^32^ and are therefore included for comparison.

**Table 1:** Chemical Formulas, Space Groups, Evaluated Surfaces, and Slab Model Details for the 19 Selected Materials

materials	space group	facet	model size
RuO_2_	*P*4_2_/*mnm*(136)	110	4 × 2, 192 atoms
IrO_2_	*P*4_2_/*mnm*(136)	110	4 × 2, 192 atoms
β-MnO_2_	*P*4_2_/*mnm*(136)	110	4 × 2, 192 atoms
γ-MnO_2_	*C*2/*m*(12)	001	2 × 1, 156 atoms
Fe_3_O_4_	Fd3̅m(227)	111	2 × 2, 168 atoms
Co_3_O_4_	Fd3̅m(227)	111	2 × 2, 168 atoms
CeO_2_	Fm3̅m(225)	111	3 × 3, 108 atoms
LaNiO_3_	Pm3̅m(221)	001	4 × 4, 240 atoms
LaNiO_3_	R3̅c(167)	0001	1 × 1, 120 atoms
LaCuO_3_	Pm3̅m(221)	001	4 × 4, 240 atoms
LaCuO_3_	R3̅c(167)	0001	1 × 1, 120 atoms
NiFe_2_O_4_	Fd3̅m(227)	111	1 × 1, 168 atoms
CoFe_2_O_4_	Fd3̅m(227)	111	1 × 1, 168 atoms
SrCoO_3_	Pm3̅m(221)	001	2 × 2, 60 atoms
PrBaCo_2_O_6_	*P*4/*mmm*(123)	001	1 × 1, 76 atoms
β-CoOOH	R3̅m(166)	0001	3 × 3, 108 atoms
β-NiOOH	*P*2_1_/*c*(14)	001	2 × 2, 192 atoms
β-NiOOH:Fe	*P*2_1_/*c*(14)	001	2 × 2, 192 atoms
γ-FeOOH	Cmc2_1_(36)	010	3 × 2, 96 atoms

### Calculation of V_O_ Formation Free Energy

The V_O_ formation free energy (Δ*G*_f_^V_O_^) on the catalyst surface was calculated using the following equation:

$$\Delta G_{f}^{V_{O}}=G_{\mathrm{slab}}^{V_{O}}+\mu_{O}-G_{\mathrm{slab}}$$ 1

where *G*_slab_^V_O_^ and *G*_slab_ are the free energy of the surface with and without V_O_, respectively. μ_O_ is the chemical potential of oxygen, given by

$$\mu_{O}=\frac{1}{2}G_{O_{2}}$$ 2

Due to the well-known limitations of semilocal functionals in accurately predicting the binding energy of O_2_, the Gibbs free energy of O_2_ was calculated relative to liquid water and hydrogen gas using the experimental formation free energy (Δ*G*°_H_2_O(l)_^exp^ = – 2.46 eV) of liquid water.

The Gibbs free energy of O_2_ is expressed as

$$G_{O_{2}}=2G_{H_{2}O}-4\left(G_{H^{+}}+G_{e^{-}}\right)-2\Delta {G^{{}^{\circ}}}_{H_{2}O\left(l\right)}^{\exp}$$ 3

Using the computational hydrogen electrode model,^33^ the relationship between the Gibbs free energy of hydrogen, pH and external potential can be expressed as

$$G_{H^{+}}+G_{e^{-}}=\frac{1}{2}G_{H_{2}}-eU_{\mathrm{SHE}}+k_{b}T\left(\ln\left[a_{H^{+}}\right]\right)=\frac{1}{2}G_{H_{2}}-eU_{\mathrm{RHE}}$$ 4

Therefore, μ_O_ can be rewritten as

$$\mu_{O}=G_{H_{2}O}-\Delta {G^{{}^{\circ}}}_{H_{2}O\left(l\right)}^{\exp}-G_{H_{2}}+2eU_{\mathrm{RHE}}$$ 5

Combining eqs 1 and 5, the V_O_ formation free energy as a function of the potential at reversible hydrogen electrode (RHE) scale is

$$\Delta G_{f}^{V_{O}}=G_{\mathrm{slab}}^{V_{O}}+G_{H_{2}O}-G_{\mathrm{slab}}-G_{H_{2}}-\Delta {G^{{}^{\circ}}}_{H_{2}O\left(l\right)}^{\exp}+2eU_{\mathrm{RHE}}$$ 6

Similarly, the chemical potential of the hydroxyl (μ_OH_) can be defined as

$$\mu_{\mathrm{OH}}=G_{H_{2}O}-\frac{1}{2}\Delta {G^{{}^{\circ}}}_{H_{2}O\left(l\right)}^{\exp}-\frac{1}{2}G_{H_{2}}+eU_{\mathrm{RHE}}$$ 7

Thus, the formation free energy of OH can then be expressed as

$$\Delta G_{f}^{V_{\mathrm{OH}}}=G_{\mathrm{slab}}^{V_{\mathrm{OH}}}+G_{H_{2}O}-G_{\mathrm{slab}}-\frac{1}{2}G_{H_{2}}-\frac{1}{2}\Delta {G^{{}^{\circ}}}_{H_{2}O\left(l\right)}^{\exp}+{eU}_{\mathrm{RHE}}$$ 8

The terms *G*_H_2_O_ and *G*_H_2__ represent the Gibbs free energy of liquid water and hydrogen gas, respectively, calculated using the atomic simulation environment (ASE).^34^

For the solid phases, zero-point energy and integrated heat-capacity contributions were neglected because their effects are marginal at room temperature.^35,36^ Solvent effects were not included because test calculations on RuO_2_ indicate that the inclusion of explicit H_2_O molecules changes the computed V_O_ formation energy by only ∼ 0.10 eV, suggesting that solvation has only a minor influence on the calculated V_O_ formation energies.

## Supplementary Material



## Notes

The Supporting Information (SI) includes calculated oxygen vacancy formation free energies referenced to gas phase O_2_ at *U*_RHE_ = 0 V; calculated oxygen vacancy formation free energies as a function of applied electrode potential for 15 metal oxides; calculated oxygen and hydroxyl vacancy formation free energies as a function of applied electrode potential for four metal hydroxides. All structural models are available in figshare (https://figshare.com/account/articles/31566304) (PDF)
