# Supplementary material for: A Thermodynamic Framework for Predicting Oxygen Vacancy Formation Energies on Electrocatalyst Surfaces
Source: ACS Omega. 2026 Jun 10;11(24):36151–6. doi: 10.1021/acsomega.6c03286 (PMC13294908; doi:10.1021/acsomega.6c03286)
Supplement: Supplementary file 1 [file ao6c03286_si_001.pdf]

Supporting Information

A Thermodynamic Framework for Predicting Oxygen Vacancy Formation Energies on Electrocatalyst Surfaces

Yuefeng Zhang[1], Zhenbin Wang[1,2*]

[1]Department of Materials Science and Engineering, City University of Hong Kong, Hong Kong SAR, 999077, China

[2]School of Energy and Environment, City University of Hong Kong, Hong Kong SAR, 999077, China

E-mail: zwan22@cityu.edu.hk

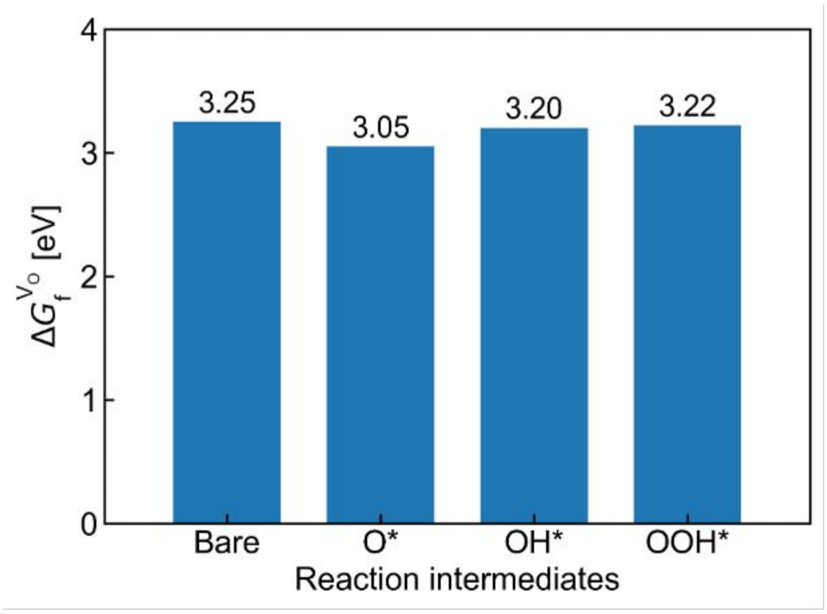

Figure S1. Calculated V_O formation energies of IrO_2 under different states, including bare, O*, OH*, and OOH*

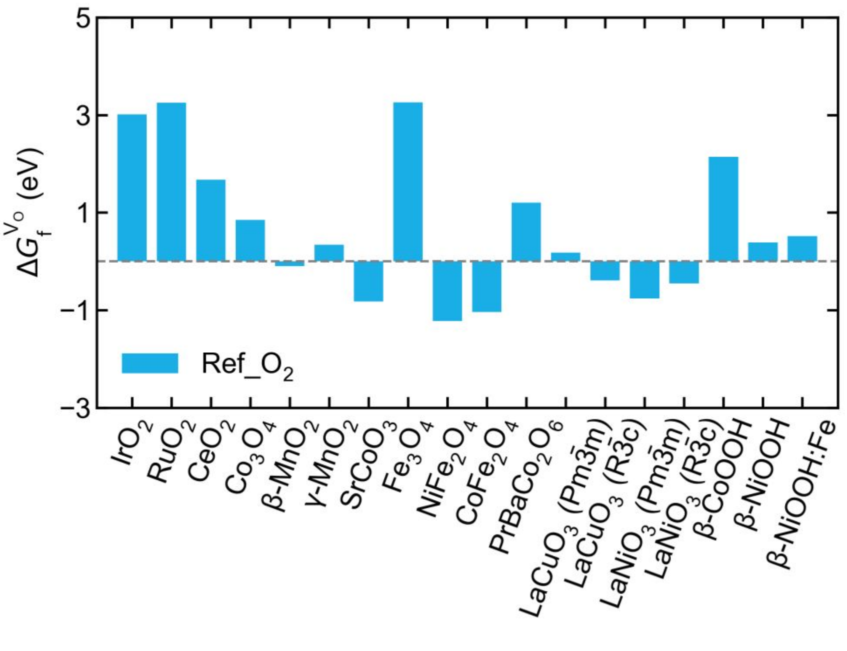

Figure S2. Calculated oxygen vacancy formation free energies ($\Delta G_{f}^{V_O}$) for 18 metal oxides and metal hydroxides, referenced to gas phase O_2 at U_{RHE} = 0 V.

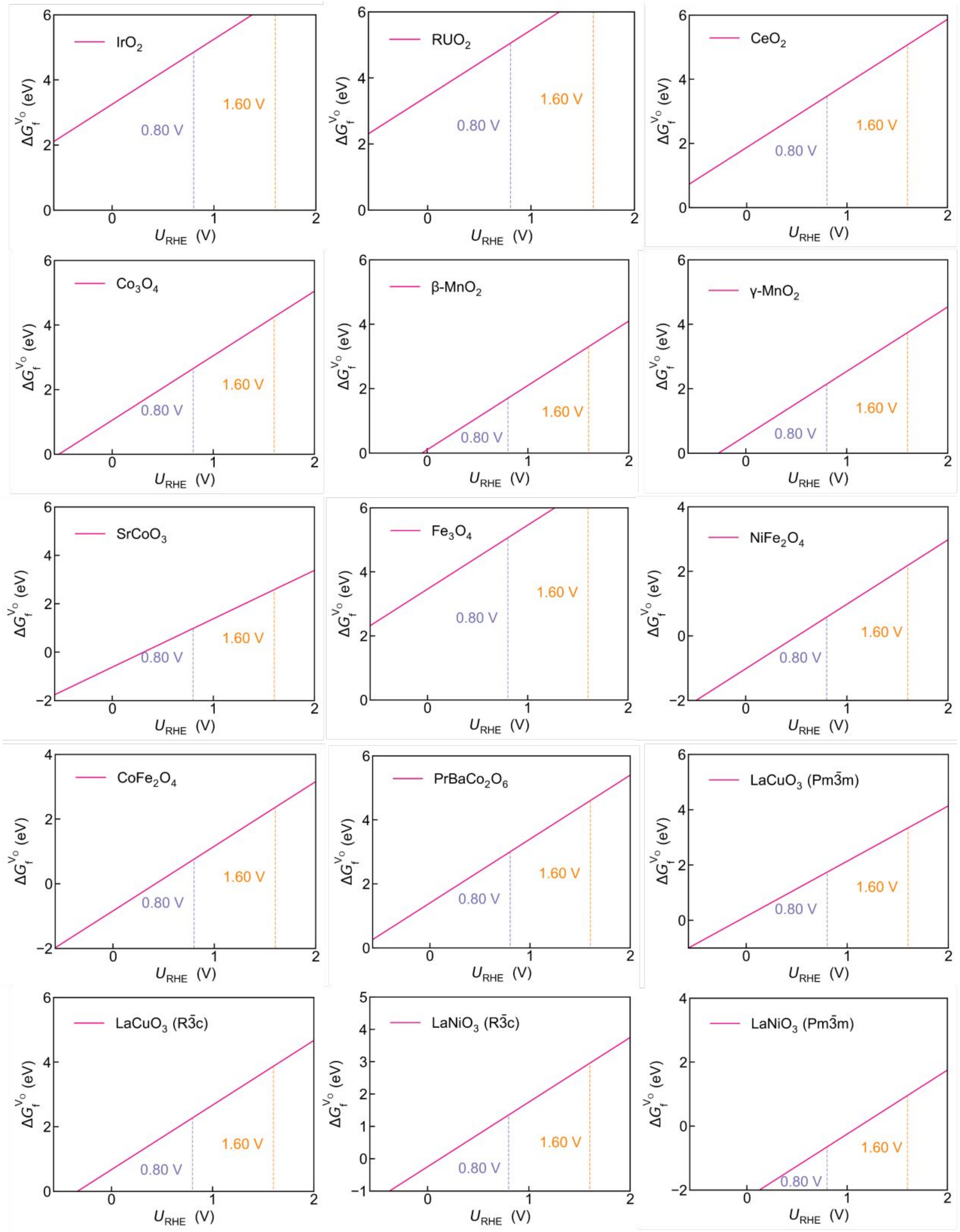

Figure S3. Calculated oxygen vacancy formation free energies ($\Delta G_{\mathrm{f}}^{\mathrm{V_O}}$) as a function of applied electrode potential (U_{RHE}) for 15 metal oxides.

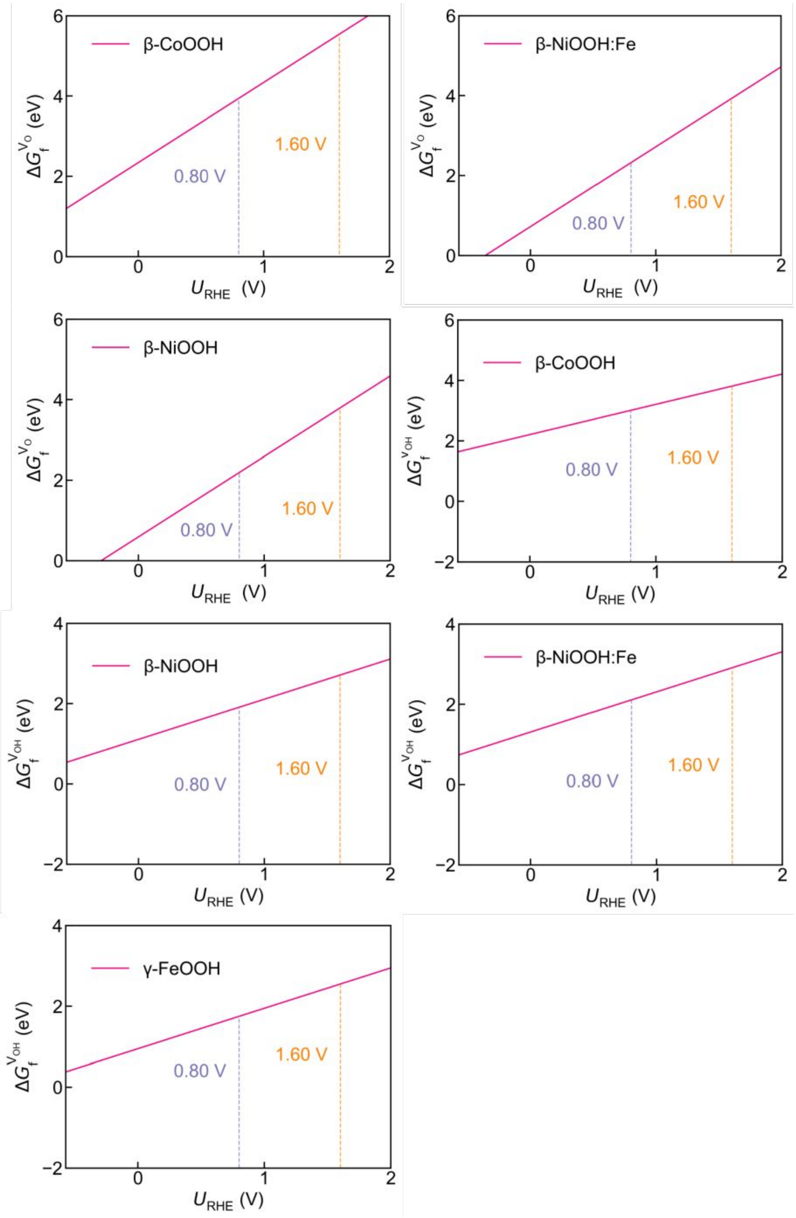

Figure S4. Calculated oxygen (O) and hydroxyl (OH) vacancy formation free energies ($\Delta G_f^{V_O}$ and $\Delta G_f^{V_{OH}}$, respectively) as a function of applied electrode potential (U_{RHE}) for all metal hydroxides.

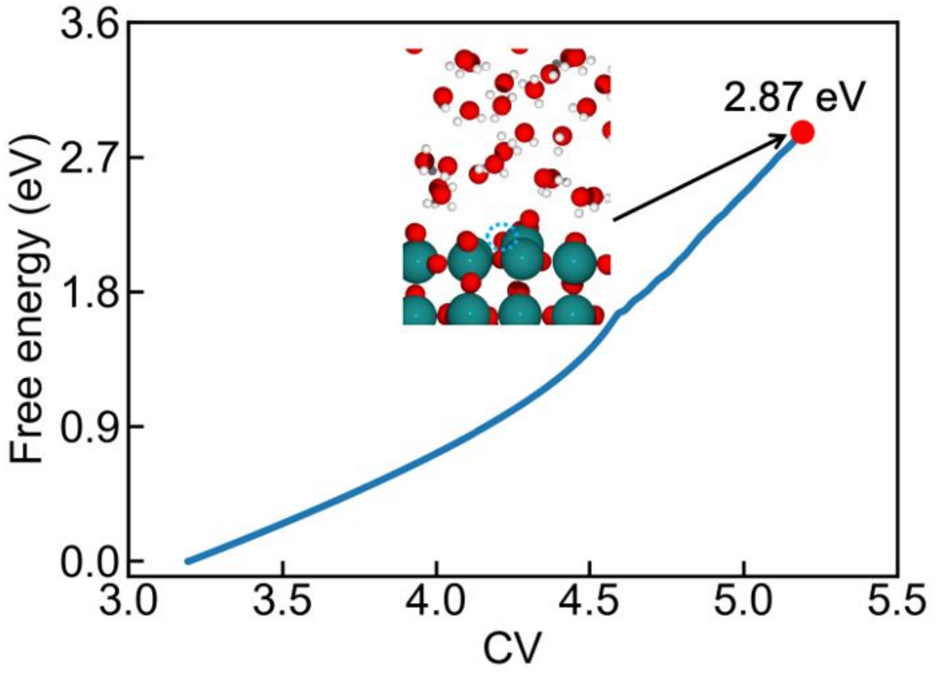

Figure S5. The calculated free energy barrier for O dissolution from the RuO_2 (110) surface using the AIMD blue moon ensemble approach. The spilled-over O is highlighted by the blue dashed circle.
